# Factors influencing the adoption of a healthy eating campaign by federal cross-sector partners: a qualitative study

**DOI:** 10.1186/s12889-016-3523-x

**Published:** 2016-08-30

**Authors:** Melissa Anne Fernandez, Sophie Desroches, Mylène Turcotte, Marie Marquis, Joëlle Dufour, Véronique Provencher

**Affiliations:** 1 Institute of Nutrition and Functional Foods, Université Laval, Pavillon des Services, 2440 Hochelaga Blvd., Quebec, QC G1C 0A6 Canada; 2 School of Nutrition, Université Laval, Pavillon Paul-Comtois, 2425, rue de l’Agriculture, local 1122, Quebec, QC G1V 0A6 Canada; 3 Department of Nutrition, Faculté de médecine, Université de Montréal, CP 6128, succursale Centre-ville, Montreal, QC H3C 3J7 Canada; 4 Department of Anthropology, Université Laval, Pavillon Charles-De Koninck, bureau 3431, 1030, avenue des Sciences-Humaines, Québec, QC G1V 0A6 Canada

**Keywords:** Adoption, Compatibility, Cross-sector partners, Diffusion of innovations, Nutrition, Relative advantages, Social norms, Qualitative study

## Abstract

**Background:**

The *Eat Well Campaign* (EWC) was a social marketing campaign developed by Health Canada and disseminated to the public with the help of cross-sector partners. The purpose of this study was to describe factors that influenced cross-sector partners’ decision to adopt the EWC.

**Methods:**

Thematic content analysis, based primarily on an *a priori* codebook of constructs from Roger’s *diffusion of innovations decision process model*, was conducted on hour-long semi-structured telephone interviews with Health Canada’s cross-sector partners (*n* = 18).

**Results:**

Dominant themes influencing cross-sector partners’ decision to adopt the EWC were: high compatibility with the organization’s values; being associated with Health Canada; and low perceived complexity of activities. Several adopters indicated that social norms (e.g., knowing that other organizations in their network were involved in the collaboration) played a strong role in their decision to participate, particularly for food retailers and small organizations. The opportunity itself to work in partnership with Health Canada and other organizations was seen as a prominent relative advantage by many organizations. Adopters were characterized as having high social participation and positive attitudes towards health, new ideas and Health Canada. The lack of exposure to the mass media channels used to diffuse the campaign and reserved attitudes towards Health Canada were prominent obstacles identified by a minority of health organizations, which challenged the decision to adopt the EWC. Most other barriers were considered as minor challenges and did not appear to impede the adoption process.

**Conclusions:**

Understanding factors that influence cross-sector adoption of nutrition initiatives can help decision makers target the most appropriate partners to advance public health objectives. Government health agencies are likely to find strong partners in organizations that share the same values as the initiative, have positive attitudes towards health, are extremely implicated in social causes and value the notion of partnership.

## Background

In 2008, nearly 63 % of deaths worldwide were attributed to noncommunicable diseases and the global burden from cardiovascular disease, diabetes, cancer and respiratory disease is expected to increase over the coming years [1]. A large proportion of these diseases are preventable with lifestyle changes including a healthy diet, yet there has been very little success in diminishing rates of diet-related chronic diseases [1, 2]. Complex societal health concerns such as obesity are influenced by a wide range of environmental and individual factors that demand collaborations between government, industry and civil society [1, 3]. Major health agencies in the US and Canada have recognized that leveraging the resources and the power of stakeholders (including influencers in private and public sectors) through partnerships is essential to shift the paradigm of poor eating patterns [4, 5]. Complex problems require multi-faceted solutions and to reverse the increasing burden of noncommunicable diseases multisectoral efforts are necessary [2, 6].

There are evident financial incentives for governmental public health agencies to engage in partnerships. During fiscal restraint, leveraging resources and expertise through partnerships becomes an attractive mechanism for governments to address complex issues such as obesity and chronic disease prevention [7]. Multisector or cross-sector partnership benefits for governments include greater reach through access to new networks, sharing resources and technology, increasing potential for innovation, leveraging resources and expertise and greater consistency in health messages through a concerted effort [2, 8]. The motivations for private-sector partners to get involved in public health initiatives include: corporate and social responsibilities, demonstrate positive public goodwill, appear more attractive to future employees, build goodwill among current staff and create additional business and profits [9]. Despite the budding potential for win-win cross-sector partnerships, there are demonstrable [10] and potential [9] conflicts of interest that need to be considered and managed carefully as not to undermine public health goals.

Little is known about cross-sector partners’ motivations for adopting healthy eating initiatives. The RE-AIM evaluation framework (reach, effectiveness, adoption, implementation and maintenance) examines adoption in terms of the number or percentage of sites that participate in a program and is often a quantitative dimension indicative of representativeness [11–13]. A purely quantitative examination of adoption, however, falls short by not describing the factors leading to adoption. Given that understanding how the adoption of an intervention can vary significantly between modalities and this can impact the intervention itself [14], it is particularly important to garner a strong understanding of the adoption processes between partners from different sectors through qualitative examination. Understanding the factors influencing adoption is expected to help inform decision makers about the most effective partners with the greatest potential for impacting public health goals and minimizing conflicts of interest. Qualitative adoption data can be used to identify and target future adopters with the best systems-organization fit. This information has potential to be extremely valuable for developing strategies for purposive targeting of partners and enhancing adoption rates [15, 16].

Child overweight and obesity was declared a public health priority in 2010 by Federal, Provincial and Territorial Ministers of Health in Canada [17]. In response to this declaration, three phases of a healthy eating and education awareness initiative were designed by Health Canada (HC). The *Eat Well Campaign*: *Food Skills* (EWC) was the third initiative; it leveraged the resources and expertise of cross-sector partners to diffuse a fully integrated social marketing campaign. Partners adopting the EWC included collaborators from the retail food industry, advertising, the media, Federal, Provincial and Territorial governments and non-governmental organizations (NGO). The purpose of this study was to describe, among cross-sector partners, the factors influencing the decision to adopt the EWC.

## Methods

### Study design

Adoption of the EWC by HC’s cross-sector partners was investigated as part of a process and impact evaluation of the EWC. Qualitative data collection and analysis was conducted to describe the factors that influenced cross-sector partners’ decision to adopt the EWC. Constructs and keywords from Rogers’ *Innovation-decision process* model that lead to knowledge generation about an innovation and persuade an organization to make the decision to adopt an innovation (i.e. the EWC) were used as a basis for describing the adoption process of the EWC. The main constructs examined were prior conditions, characteristics of the adopter and characteristics of the innovation [18] (Table 1).

**Table 1:** Themes based on Rogers’ *Innovation-decision process model* [18] influencing the decision to adopt the *Eat Well Campaign: Food Skills* (EWC)

Parent themes and subthemes	Definitions
Prior conditions	All prior experiences, perceptions and attitudes that can shape the organization’s knowledge about the EWC and persuade them to adopt it
*Previous practice*	Any organizational experiences that can help create knowledge about the EWC
*Innovativeness*	The organization’s perception of the EWC as a new idea
*Norms of the social system*	Perceptions of practices and behaviors that the organization is expected to conduct in relation to the EWC. These norms are set by the organizations’ social network (peers, clients, public, audience, etc.)
*Perceived need or problem*	The recognition of the organization’s internal need or problem that can be addressed by adopting the EWC
Characteristics of the adopter^a^	Any characteristic of the decision making unit (i.e. organization) that will shape their knowledge and attitudes towards the EWC
*Communication behaviour*	Descriptors of the organizations’ internal and external communication style, habits, exposure to media and involvement in social and public networks
*Personality variables*	Human personality characteristics perceived to be associated with or attributed to the organization
Characteristics of the innovation^b^	Characteristics of the EWC perceived by the organizations that may persuade them to adopt it
*Compatibility*	The perception by organizations that the EWC is consistent with their existing values, practices, experiences or needs
*Complexity*	The perception by organizations that the EWC is difficult to understand or implement
*Relative advantages*	The perception by organizations that the EWC is better than potential alternatives and can be measured in terms of benefits

^a^Socioeconomic characteristics is the final characteristic of the decision making unit, but none emerged and therefore this subtheme was not included

^b^Triability and observability are the other two characteristics of the innovation that are commonly investigated; however, they were not relevant to the EWC and these subthemes were not included

### Data collection

**Fig. 1:**
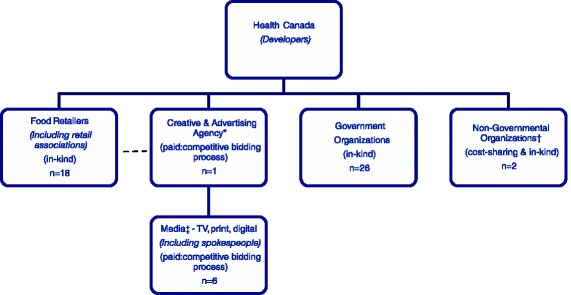
The *Eat Well Campaign: Food Skills* collaboration. * The creative and advertising agency was an intermediary between individual food retailers, the media and Health Canada. † For reporting purposes, Non-Governmental Organizations were combined with Government Organizations and were collectively called “health organizations”. ‡ For reporting purposes, media partners were combined with the creative and advertising agency and were collectively called the “media”

With HC assistance 37 of the 53 partners involved in the EWC collaboration were purposefully selected and invited to participate in the study (Fig. 1). Purposeful selection was based on role, partner-type and timing of involvement in the EWC. Health Canada provided contact information for key informants at each organization invited to participate in the study, but one organization that was not actively engaged in the EWC at the time of study conception was not invited. Key informants were invited to participate in the study by e-mail, telephone or both. During the first round of interviews a purposeful sample of 24 partners were invited to take part in the study. Those who agreed to participate were provided with confidentiality agreements and gave their informed consent. An interview guide and a brief web-questionnaire asking participants to describe their organizations’ involvement in the EWC were provided. An hour-long semi-structured interview based, in part, on Roger’s *Diffusion of innovations* theory [18] was administered over the phone by a bilingual interviewer (M.T.). To complete suspected data gaps identified during the first round of interviews, 13 additional participants were purposefully selected from the food retailer and the health organization groups. Additional interviews did not provide new or different information. Given that the objective of this study was to describe factors influencing the decision to adopt the EWC, participants from HC’s own regional offices were excluded (*n* = 3). Fifteen partners refused to participate, one participant dropped out and a total of 21 interviews with partners were conducted, 3 were excluded and the remaining 18 interviews were analyzed: *n* = 8 food retailers; *n* = 6 media; *n* = 7 health organizations.

### Data analysis

Interviews were digitally recorded and transcribed verbatim. Transcript quality was verified (by M.A.F. or J.D.) to ensure language accuracy and avoid misinterpretation. Transcripts were coded by three bilingual research assistants (M.T., M.A.F., J.D.). Thematic content analysis was conducted on transcripts using Saldaña’s [19] method of first and second cycle coding to first categorize and then interpret data. NVivo software (version 10; QSR International, Doncaster, Victoria, Australia) was used to organize data. A deductive-inductive data analysis approach was employed whereby excerpts were initially lump coded into an *a priori* codebook followed by the inductive splitting of subthemes into micro-themes. The codebook was based on the *Innovation-decision process* model [18] and interview question keywords. All three coders were involved in the first round of lump coding. Coding agreement between coders was over 80 % and was verified by triple coding five interviews. Codes were split during subsequent rounds of coding and code interpretation. Code splitting was conducted separately by two coders (M.T. and M.A.F) and then validated as a group. French excerpts were translated into English by an Anglophone (M.A.F.) and verified for accuracy by a Francophone (J.D). Validation was done by verifying procedures and methods with senior researchers (S.D., M.M., V.P.) during peer debriefing sessions [20]. Contextual coding was carried out throughout the data analysis process to ensure organizational context was maintained.

## Results

### Adoption of the EWC by cross-sector partners and decision making context

Health Canada invited a very large retail association (Retail Association 1) and an independent food retail association (Retail Association 2) to participate in the EWC. Both retail associations agreed; all 8 food retail members from Retail Association 1 and 8 members from Retail Association 2 adopted the EWC. Recruitment of Retail Association 2 members into the EWC was done through a third party and non-adopters remained anonymous to HC. All paid collaborators (media partners) that were invited adopted the EWC. Among health organizations, all 13 Canadian Provinces and Territories adopted the EWC as well as Federal health organizations who were already involved in working groups (the Federal/Provincial/Territorial Group on Nutrition and the Healthy Eating and Education Awareness Initiatives Task Force) that were mandated to support the EWC. In addition, the 2 non-governmental health organizations who were invited to collaborate adopted the EWC. Several respondents reported that the decision itself to adopt the EWC campaign was made relatively easily, without hesitation.

“I think we kinda assumed that we had to do it, like it wasn’t an option to not do it is the way I saw it, because, well it’s a retailer campaign and we’re a big retailer so we wouldn’t really not do it, I guess.” *- Food retailer*

Other contextual factors surrounding the decision to participate involved: collaborative discussions with HC; a way to demonstrate corporate citizenship; financial influences (both investments made by in-kind partners and income received by paid-partners); opportunities to support HC and support good messages.

### Description of the participating organizations and their roles

Table 2 provides a description of the characteristics of the 18 organizations who participated in the study as reported by key informants. Adopters of the EWC consisted of three types of partnerships; paid, in-kind (volunteer) and cost-sharing. However, some paid and in-kind contractors also saw themselves as cost-sharing collaborators.

**Table 2:** Characteristics of participating organizations reported by key informants

Characteristic	Frequency (*n*)	Percent (%)
Type of partner^b^		
Retail food industry (retailers and retail associations)	8	44
Communications (media, advertising, and spokespeople)	6	33
Health organizations (NGO, provincial and territorial)	4	22
Perceived type of relationship		
Paid contractor	6	33
Unpaid volunteer (in-kind)	2	11
Cost-sharing collaborator	6	33
Both paid-contractor and cost-sharing collaborator	1	6
No response	3	17
Head office location^a, b^		
West Coast or Prairies	3	17
Central Canada	12	67
Maritimes or Far North	3	17
Regional activity^a, b^		
National	7	39
Most Provinces and Territories	2	11
West Coast and Prairies	2	11
Central Canada	4	22
Maritimes or Far North	3	17
Active outside of Canada		
Yes	3	17
No	15	83
Organizational size		
Small (fewer than 50 employees)	2	11
Medium (between 50 and 250 employees)	2	11
Large (over 250 employees)	14	78
Congruency of organizational mission with healthy eating or healthy lifestyles		
Yes	14	78
No	3	17
No answer	1	6

^a^Regional definitions: West Coast, British Columbia; Prairies, Alberta, Saskatchewan and Manitoba; Central Canada, Ontario and Quebec; Maritimes, Newfoundland and Labrador, New Brunswick, Nova Scotia and Prince Edward Island; Far North, Yukon Territory, Northwest Territory and Nunavut

^b^Some groups were combined to maintain the confidentiality of easily identifiable participants

“Well I think that because Health Canada was funding the development of the artwork and they helped fund a lot of base costs and then we also paid for production and materials and distribution and, you know, added support and staff and that type of thing. So it was a jointly funded program.” *– Food retailer*

One paid contractor perceived themselves as true partners with HC in the EWC, because of their perception of cost-sharing. There was a semblance of pride linked to the idea of being a partner and not just contractor.

“We weren’t just diffusers; we were real partners in this cause.” *- Media*

Other specific roles that were identified by some partners included: diffusers of information, producers of material, information sharing with HC, intermediaries with other organizations or the public, spokespeople and message translators.

“Our role was to translate the message from Health Canada into a more accessible form for average people and families at home to give them recipes, to inspire them and to really get them on the right path to making better food choices for their families.” *- Media*

### Prior conditions: previous practice

All partners interviewed had previous experience with healthy eating, healthy lifestyle or community campaigns. The majority of partners had no direct experience working with HC although this did not appear deter their decision to participate, whereas having had previous experience working directly with HC appeared to facilitate the decision to adopt the EWC.

“We had worked with them before, you know we had that already… the relationship developed, and so we didn’t have to worry about worrying, you know about things like oh, how will they react or whatever, like we knew what to expect from them.” - *Media*

### Prior conditions: innovativeness

A slight majority of participants across partner types did not perceive the EWC as innovative mainly because the theme and messages were not novel. It was seen as just another education campaign and not different or exciting.

“I felt that they were messages that I’ve seen a million times before delivered through other campaigns by other organizations. I didn’t think it was unique to Health Canada, you know.” *- Food retailer*

Despite the lack of perceived innovation by most partners, the campaign was considered relevant to the public. Some partners considered the approach HC used to make federal health messages accessible to the public as innovative. The quality of the graphics and materials, the use of many diffusion channels, the use of point of purchase messaging and the combination of traditional and social media were also viewed as innovative aspects of the EWC by a few partners. In addition, the cross-sector partnership itself was considered as an innovative way for HC to diffuse a healthy eating initiative by a food retailer and members of health organizations.

“Oh! Yeah, I think that, again it’s the collaboration that was unique about this - multiple retailers all at once communicating a message.” *– Food retailer*

### Prior condition: norms of the social system

Many respondents spoke about social norms influencing their decision to adopt the EWC. All respondents who spoke about social norms were food retailers or worked for a small organization. The primary influence for major retailers was their professional network, Retail Association 1. For these respondents, there was a sense of wanting to be part of the group and a minor sense of peer pressure to follow in line with their peers/competitors.

“Well, it was a campaign that brought together the majority of retailers, you know. So, we knew that our competitors would probably be participating and we didn’t want be left out.” *– Food retailer*

Respondents from small organizations spoke about how the public expected them to be involved in this type of initiative. One in-kind respondent mentioned that while their social network was important, they would have participated regardless of other organizations, because it was “the right thing to do”.

### Prior conditions: perceived need or problem

The majority of organizations spoke about the EWC meeting needs and problems of their clients rather than their own organization. This information was captured under “relative advantages” for organizations. No organizational problems were mentioned during interviews. The EWC, however, addressed a few organizational needs; a food retailer mentioned the need to educate their clients about healthy eating; a health organization spoke of the need to raise awareness about healthy eating within their region; and a food retailer mentioned the need to be involved in activities that support their position as a leader in healthy eating.

“The important part is for us to raise awareness about healthy eating. […] food is very much a part of our culture, and historically some of our choices are not what we would consider healthy.” *– Health organization*

### Characteristics of adopters: communication behavior

Interconnectedness played a major role in the adoption process of the partners. The majority of participants were recruited through established networks; Retail Association 1, Retail Association 2 and the Federal/Provincial/Territorial Group on Nutrition. It was through these usual social networks that participating organizations came to know of the campaign. In addition to these professional networks, interconnectedness within large organizations (between departments) as well as between organizations and the government were identified. A notable communication barrier that emerged was the lack of exposure to mass media by a partner from a health organization that was not located in Central Canada. This limited their exposure to the EWC and was seen as an obstacle that seriously challenged its adoption. The notion of high social participation organically emerged from most food retailers and media respondents who described having notable experiences working with charities, in other social causes, fundraising, etc.

“It would take me like hours to tell you about all the things that we do in community to teach kids how to eat healthy. You know from getting out, going to schools and having kids coming to our schools and hiring dietitians to talk to kids” *– Food retailer*

### Characteristics of the adopter: personality variables

The organizational personality traits identified were favorable attitudes, empathy and a strong level of rationality. Rationality emerged as an organizational personality variable related to the decision to adopt the EWC by half the respondents.

“A core essence of what we’re about is helping Canadians […], and so a program that helps support our overall arching objective of helping people to understand that direct relationship between the food they eat and how they feel and how they live is a benefit.” – *Food retailer*

Favorable attitudes towards health, new ideas and HC were seen as organizational personality traits that influenced the adoption of the EWC by all partners except two health organizations. These two partners held reserved attitudes towards HC, which appeared to be obstacles challenging their adoption of the EWC. A high level of empathy was demonstrated towards the EWC target population by a small number of partners in the food retail group and towards HC by a media partner.

“We felt also that Health Canada had a very strong message but they also didn’t really have, you know, a huge budget to accomplish this. So, if this was not Health Canada, for us it probably wouldn’t have been worth our while, but we wanted to make the effort because we think that it’s a good message […] So, we really made an effort to, you know, meet all the requirements they had.” *- Media*

### Characteristics of the innovation: compatibility

One health organization and one food retailer spoke of the lack of compatibility between the EWC and their organizations, whereas among the remaining partners there appeared to be a very strong sense of fit with the EWC. Partners spoke of sharing similar values, mission or vision with the EWC and/or HC. Half of the food retailers and some media partners described their organizations as having very different missions, but sharing the same values as the EWC and HC. A media partner and a health organization spoke of sharing the same mission as HC but having extremely different visions. Sharing the same values appeared to be more important than having the same (public health-oriented) mission or vision on how to address health and nutrition.

“Because we are a media company […] our mandate is to entertain and not really to educate the public on healthy lifestyles. It’s a cause that we consider important, that we think is noble like I mentioned before, but this doesn’t mean that it’s a cause that our organization supports systematically.” *- Media*

The fit between the organizations’ practices, target population and the EWC was also important. Some media partners spoke of a strong fit between their brand and clients with the activities and target population of the EWC. Partners from all groups described having the same audience or clientele as the EWC.

“Just tips for families ‘cause that’s really our demographic right, it’s families that are on the run and on the go and… So the campaigns really seemed to fit with the timing.” *– Food retailer*

An emergent element from a small number of food retailers and media partners was staff fit between organizations’ employees and the EWC. Poor staff fit was identified as a challenge for one food retailer, but did not impede organizational adoption, whereas compatibility between employees’ personal values, beliefs and work ethic with elements of the EWC facilitated adoption.

“So, it’s a lot of work goes into, you know. I’m the type… I don’t just wanna take a poster and stick it up on a wall.” *– Food retailer*

### Characteristics on the innovation: complexity

Overall, most partners did not perceive that adoption implicated much complexity. A handful of respondents across partner groups perceived no complexity whatsoever, because of past individual experiences, simple messaging and dissemination. Across partner groups, minor reasons for the EWC being considered as a little or somewhat complex included: problems related to planning and task management, political issues, limited financial resources, strict control of information by HC, lack of relevance to population groups, lack of clear objectives, lack of familiarity with marketing, difficult messaging to transmit and difficulties integrating activities within the organization.

“I initially I found it hard to sort of understand what they were trying to accomplish. And maybe it was because, you know, you’ve got the big forum and the big conference call and that and that’s where I found that it was hard to understand at the very beginning as to what exactly what they were doing and maybe I wasn’t in the ground level to really understand and that’s what my perception was.” *– Food retailer*

### Characteristics of the innovation: relative advantages

Having emerged entirely organically, the most salient relative advantage of the EWC for partners was the impression that it could enhance their image and credibility. This relative advantage was identified by all members of the food retailer group as well as a few members from other partner groups.

“Well I think it’s partnering with a credible organization, so we are trying… like our goal is to demonstrate to our customers that we are committed to health and wellness. And, by partnering with a credible organization like Health Canada, it’s… you know, positive for us.” *– Food retailer*

In addition, being associated with HC was a prominent relative advantage for all partner groups. The partnership aspect of the EWC was also a prominent relative advantage across partner groups for in-kind, paid and cost-sharing collaborators.

“Yeah, I think a coordinated effort is certainly preferred as opposed to… you know we may not have gotten involved as an individual company in the Eat Well Campaign specifically, just because we have lot of other things going on as well in the same areas as far as communicating health messages. So, that was an important part of it.” *– Food retailer*

A common relative advantage for all food retailers and some other partners was the EWC’s ability to respond to the nutritional needs and problems of their clients/audience as well as provide them with relevant information. Economic advantages were identified by paid and cost-sharing collaborators as well as a very small number of in-kind partners. The opportunity to increase the reach or visibility of their organization and its products through the EWC was identified by all types of partners. Obtaining new health content and materials was a relative advantage for a very small number of organizations across partner groups. The nature of the campaign itself (positive messaging and health promotion) emerged as an interesting relative advantage, particularly for media partners.

“It is healthy lifestyles, after all. It’s a great cause. It’s understood that yes, there is revenue coming in, there is an advertising investment associated with this, but we always prefer to work on projects like this one, in partnership, when it’s a good cause.” *- Media*

For some food retailers, the EWC supported their organizational values. A food retailer also mentioned benefits for the organizations’ staff around healthy eating awareness.

“Whenever we do a program like this, we don’t just offer it to the customers. We also send it out to all of our employees as well” *– Food retailer*

The majority of respondents across partner groups did not perceive any disadvantages of adopting the EWC. A media partner described challenges around communicating the actual messaging. Minor disadvantages identified by half of the food retailers were related to the financial costs of participating (investments in publicity and human resources).

“Right, because often times vendors will pay money to be in your flyer. So, you know, if you take away products that you’re gonna make money on to put in an Eat Well Campaign, that you don’t know if you’re gonna make any money on, then, you know, that would be one of the challenges.” *- Retailer*

## Discussion

### Overall perceptions

Partners had a very positive attitude towards the adoption of the EWC and the partnership with HC. Many facilitating factors emerged from the interviews whereas very few barriers associated with adoption were mentioned, despite prompting. Few perceived barriers of the EWC by adopters might be explained by HC’s targeted approach selecting partners through networks with high opinion leadership value [16]. This invitation-based approach may have reduced the potential to engage in collaborations with unfavorable partners or those with low compatibility. This is also supported by the findings that there were extremely high perceptions of fit and favorable attitudes towards health, new ideas and HC. Furthermore, the feel-good nature of the campaign may have attracted fully engaged partners that had little to no reservations for adopting a healthy eating initiative as evidenced by the easy decision making by most organizations. Uncovering barriers to adoption is particularly important in non-adoption settings [21]. Given that this study focused on factors related to the decision to adopt the EWC and not differential adoption, it is understandable that few prominent barriers emerged among actual adopters. Nevertheless, HC speculated that the lack of resources and capacity were likely factors that prevented adoption of the EWC by the majority of non-adopters from Retail Association 2 (personal communication with HC).

Recognizing in-kind and paid collaborators as cost-sharing partners in a healthy eating initiative can lead to the valorization of their expertise. This type of simple recognition could be used to strengthen their level of commitment towards an initiative through the notion of balanced contributions [4]. The activity and main location of the organizations involved suggests that the EWC was diffused more in provinces in Central Canada (i.e. Ontario and Quebec), validating one participant’s perception about not having much exposure. The majority of organizations had over 250 employees suggesting a very large capacity for reach of the adopting organizations. Given that reach of the targeted population is strongly impacted by adoption [22], the participation of large Canadian organizations in the EWC indicates that reach is likely to be high across Canada, particularly Central Canada. While the adoption rate is unknown among the food retailer group, there appeared to be greater representation from Retail Association 1 whose members all adopted the EWC. Non-adopters were concentrated among Retail Association 2 members, but the exact number and details of their non-adoption remain unknown. Food retailers in Retail Association 1 are among the leading retail corporations in Canada [23], and their potential client reach is significant. For example, in 2012 four of their members represented 30, 15.1, 14.4 and 6.4 % of the entire retail food market in Canada, respectively [24]. Therefore, members from Retail Association 2 contributed to a small percentage of the potential reach of the EWC (based on their share of the Canadian food retail market) in comparison to the major food retail corporations in Retail Association 1.

### Facilitators

The construct of prior conditions is expected to shape an individual’s attitude and knowledge towards an innovation ultimately influencing their decision to adopt or reject it. Prior conditions are characterized by previous experience, perceived innovativeness, social norms and a perceived need or problem [18]. Having experience with HC, skills and knowledge with healthy eating, familiarity with healthy eating initiatives or experiences with other lifestyle or community engagement programs appeared to facilitate adoption. Experience with similar innovations or knowledge about a subject is known to facilitate adoption as seen in Olstad [25] who studied adoption in the context of implementing nutrition guidelines in recreational facilities. This may be particularly true for partners who had experience working with HC and were used to working within governmental constraints.

A few innovative characteristics of the EWC were identified; however, the only theme that emerged influencing the decision to adopt was the notion of cross-sector partnerships. The *Diffusion of innovations* implicates high levels of perceived innovation with early adoption [26]. In the EWC, however, the adoption of the campaign was based on the timing of an organization’s involvement making the notion of early versus late adoption irrelevant. Greenhalgh [27] also noted that the notion of early adoption was less relevant in the context of organizational adoption of an idea versus individual adoption of a product. Perceived innovativeness appeared to have a neutral influence on adoption, with the exception of partnerships. Among partners who perceived no innovation, the relevance of the campaign topic appeared to have a greater influence on their attitudes then the EWC’s innovative potential. Many partners perceived an absence of innovativeness associated with the EWC indicating that its messages have already been delivered by other organizations, all the while recognizing the value of relaying important messaging again. This suggests that innovativeness was not a relevant prior condition for the EWC. Instead, strategically timing the EWC’s appearance in a cluster of already-accepted campaigns may have endowed it with pre-conceived favorable attitudes by recycling the positive behaviors organizations previously developed towards other known innovations [16].

Social norms were extremely important for food retailers as well as small organizations. Innovations that are aligned with societal norms are more likely to be accepted [18, 28]. Professional networks such as Retail Association 1 appeared to have a major influence on the decision to adopt the EWC campaign for major food retailers. A few organizations also indicated that their clients or audiences expected them to participate in these types of initiatives indicating the significance of social image. The importance of professional networks, social image, potential peer pressure and their influence on adoption is consistent with the adoption of an innovation or policy [16]. This social reputation, particularly for small organizations, is important in defining its personality and thus perceptions about the organization [29]. Social norms did not appear to be as relevant for paid contractors suggesting that in the absence of a contractual agreement, social norms were extremely important decision making factors for cross-sector partners. Despite social networks, an in-kind partner expressed feelings of moral obligation to participate in the EWC campaign alluding to the importance of the perceived value of the cause itself in respect to social norms. Adoption is also thought to be facilitated when an innovation is perceived to have high public support [16].

An innovation’s potential to respond to a need or resolve a problem is an important prior condition influencing attitudes and knowledge about an innovation [18]. In the case of the EWC, it was not perceived as being able to resolve any problems of adopters. The EWC, however, did respond to a few organizational needs of a minority of food retailers and health organizations. Perceived needs and problems were insufficient to influence adoption for the majority of cross-sector partners, which is likely driven by other attributes such as perceived risk or the innovations-systems fit [27]. These findings suggest that factors beyond responding to their own needs may be more important in convincing some organizations to adopt health promotion collaborations. The lack of influence of the perceived needs attribute may also be related to the fact that respondents did not speak of the innovation as responding to their own organizational needs or problems, but rather general needs of the public. For example, nearly all respondents agreed that the EWC responded to their clients or the public’s needs. This attribute seemed to be a strong relevant advantage related to their decision to participate in the EWC, particularly for food retailers.

Few organizational characteristics related to adoption of the EWC by organizations were observed. Nevertheless, favorable attitudes in general towards HC, health and new ideas can be considered as significant facilitators for adoption. Furthermore, there appeared to be extremely positive attitudes towards the adoption of the EWC among organizations where there was extremely good staff compatibility. Successful partnerships and collaborations as well as capable staff have also been identified elsewhere as facilitators for adoption [30]. The favorable attitudes of respondents is similar to the finding of Olstad et al. [31] where managerial receptivity for change was a facilitator for adoption of voluntary nutritional guidelines by recreational facilities. The ability to empathize with HC or the target population was a facilitating organizational personality variable that was identified in a small number of food retailer and media respondents and is compatible with the characteristics of adopters of innovations [18].

Interconnectedness seems to have played a major role in influencing the decision to adopt the EWC. This finding is congruent with the discourse on social network theory, whereby interconnectedness is considered as central to social networks and adoption is driven by these social relationships [32]. Large organizations were well-connected to outside organizations via professional networks (e.g. Retail Association 1) and internal government relations departments. Professional networks appeared to be key trust leaders in the adopters’ decision to engage in the EWC. Connections to professional networks (Retail Association 1, Retail Association 2 and the Federal/Provincial/Territorial Group on Nutrition) facilitated the decision to adopt the EWC; organizations were often made aware of the EWC via these connections. This appeared to be particularly important for in-kind collaborators. Frequent social participation by organizations indicated that it was a common communication behavior among food retailers and the media. This finding suggests that non-health organizations who engage in social participation activities may be more inclined to engage in public health initiatives. No socioeconomic characteristics of organizations emerged as themes or subthemes during interviews. There was greater representation of adopters from large organizations in the EWC indicating that they may be more visible to government or have greater organizational ease to participate in these types of initiatives. However, this may also reflect the likelihood that larger organizations (e.g. Retail Association 1 members) were targeted more than smaller ones (e.g. Retail Association 2 members) by HC.

Compatibility, relative advantages and low complexity were dominant facilitators for the adoption of the EWC and according to Tornatzky and Klein [33] they are the most important innovation characteristics associated with adoption [33]. Furthermore, relative advantages are one of the most influential factors of adoption [28]. Relative advantages that emerged organically appeared to be the most persuasive; social prestige, association with HC and working in collaboration. The partnership aspect and idea of collaborating with HC and multiple partners was a prominent motivator for many organizations and can be considered as a strong facilitating factor. Social prestige is a subdimension of relative advantages that is known to influence the rate and extent of adoption [32]. The relative advantage of social prestige overlaps with the innovation characteristic of social norms and the organizational characteristic of interconnectedness, and appears to relate to social network theory [30]. In the case of the EWC, compatibility of the innovation with organizations’ values was a prominent characteristic that appeared to be important for nearly all adopters. This innovation-systems fit has been identified as being more important than an innovation’s actual characteristics [27]. The strategic fit between partners and the HC was a key to facilitating adoption [16] and having the same values as the EWC was particularly important and was likely to be a strong factor influencing the decision to adopt it. Fit between organizational values, organizational practices, the target audience, the staff spearheading the campaign and the EWC were also extremely important factors facilitating adoption. There is strong evidence that both the fit between values and existing practices with an innovation are related to adoption [27, 34]. Finally, the lack of perceived complexity towards the EWC speaks to a perceived ease of employing the innovation and is linked to increased probability of adopting it [28].

### Challenges

The adopters interviewed spoke of few barriers, and those that were identified were described as challenges rather than strict barriers to adoption. Notable obstacles that seriously challenged adoption were only identified among health organizations. The obstacles associated with adoption were reserved attitudes towards HC and the lack of exposure to the mass media channels used to diffuse the EWC. These obstacles were extremely important challenges given that the EWC was a social marketing campaign spearheaded by HC. Nevertheless, most other challenges mentioned by adopters were largely seen as minor factors and did not appear to dissuade adoption. For example, the extremely different visions of some partners on how to approach nutrition and health may have been a challege, but it was likley compensated for by the high compatability between organizations and the EWC. The minor presence of barriers is also likely linked to a low perception of risk versus benefits (relative advantages) [34]. The utilization of professional networks and targeting of specific partners by HC may have further reduced systematic adoption barriers for many partners. Despite financial concerns mentioned by a few partners, both in-kind and paid collaborators were willing to absorb costs associated with additional services, staffing and production expenses, whereas costs are generally a major barrier to adoption [16]. Other minor factors challenging adoption included political issues, strict control of information by HC, lack of relevant population groups, lack of clear objectives, difficulties integrating activities within the organization, etc. While these challenges did not appear to dissuade the adoption process, they are similar to others identified as having an impact on implementation [27].

### Study limitations and strengths

Non-adopters were unknown to HC, and for confidential reasons it was not feasible to seek them out and inquire about their rejection of the EWC. Nothing is known about the organizations that refused to participate; therefore, barriers to adoption are reported with an adopter’s perspective. The lack of information from non-adopters was a major limitation in the present study, leaving a knowledge gap about factors that were associated with the decision to reject the EWC. While HC speculated that non-adoption was related to fewer resources and less capacity of small organizations, conclusions are limited without actual data. On the other hand, non-adopters were comprised solely of small independent retailers and their contribution to the entire EWC collaboration and its reach was likely extremely limited in comparison to major retail corporations, media and health organizations. The results presented are perceptions from HC’s cross-sector partners that adopted the EWC and they cannot be generalized to contexts beyond governmental partnerships promoting health. The main strength of this study is the diversity of organizations providing a wide range of responses and comparisons across partner groups. The diversity also allowed analyses to approach data saturation with a relatively small number of interviews; additional respondents from the second round of interviews did not produce different or new information. Multiple-pass coding (several rounds and two cycles) adds robustness to the qualitative data analysis methodology. In addition, coding, data interpretation, procedures and methods were corroborated by at least one other person or the entire team adding validity to the results presented. This study focused on the adoption of the EWC by HC’s cross-sector partners, the primary diffusers of the campaign, but it would also be interesting to investigate the community level adoption and diffusion of the EWC by ground level intermediaries.

## Conclusion

Based on Rogers’ *Innovation-decision process* model, the main facilitators that influenced the decision to adopt the EWC by cross-sector partners were closely related to compatibility between their values, practices, target audience and the campaign. Furthermore, the social prestige of being associated with HC or a social cause led by HC was an important influencing factor. Based on the findings from this study, the key recommendations for public health organizations seeking to establish cross-sector partnerships are as follows:

1. A targeted approach for partner selection is very effective to achieve high adoption rates and may help to minimize challenges associated with the adoption of a health initiative.
2. Public health organizations can find highly compatible cross-sector partners among organizations with similar values, regardless of their mission or vision.
3. Using professional networks with strong leadership value to recruit in-kind partners is efficient.
4. A public health organization’s strong reputation can be used as leverage to attract desirable collaborators, particularly among organizations who value the notion of “working in partnership”.
5. Efforts should be made among public health organizations to be cognizant of non-adopters in order to better understand the barriers associated with contributing to health initiatives.

## Abbreviations

EWC: Eat well campaign
HC: Health Canada
NGO: Non-governmental organization
